# Automatic Distortion Rectification of Wide-Angle Images Using Outlier Refinement for Streamlining Vision Tasks

**DOI:** 10.3390/s20030894

**Published:** 2020-02-07

**Authors:** Vijay Kakani, Hakil Kim, Jongseo Lee, Choonwoo Ryu, Mahendar Kumbham

**Affiliations:** 1 Information and Communication Engineering, Inha University, 100 Inharo, Nam-gu Incheon 22212, Korea; 2 Future Vehicle Engineering, Inha University, 100 Inharo, Nam-gu Incheon 22212, Korea; 3 Valeo Vision Systems, Dunmore Road, Tuam, Co. Galway H54, Ireland

**Keywords:** automatic distortion rectification, wide-angle lens, fish-eye lens, advanced driver-assistance system (ADAS), video-surveillance, vision tasks

## Abstract

The study proposes an outlier refinement methodology for automatic distortion rectification of wide-angle and fish-eye lens camera models in the context of streamlining vision-based tasks. The line-members sets are estimated in a scene through accumulation of line candidates emerging from the same edge source. An iterative optimization with an outlier refinement scheme was applied to the loss value, to simultaneously remove the extremely curved outliers from the line-members set and update the robust line members as well as estimating the best-fit distortion parameters with lowest possible loss. The proposed algorithm was able to rectify the distortions of wide-angle and fish-eye cameras even in extreme conditions such as heavy illumination changes and severe lens distortions. Experiments were conducted using various evaluation metrics both at the pixel-level (image quality, edge stretching effects, pixel-point error) as well as higher-level use-cases (object detection, height estimation) with respect to real and synthetic data from publicly available, privately acquired sources. The performance evaluations of the proposed algorithm have been investigated using an ablation study on various datasets in correspondence to the significance analysis of the refinement scheme and loss function. Several quantitative and qualitative comparisons were carried out on the proposed approach against various self-calibration approaches.

## 1. Introduction

The usage of wide-angle camera lenses in vision-based applications demands greater precision in terms of image projection geometry such as distortion compensation and maintaining pixel consistency. There appears to be a plethora of challenges involved in the context of employing wide-angle lens models for applications such as advanced driver-assistance system (ADAS) and video surveillance.

### 1.1. Challenges

**Figure 1:**
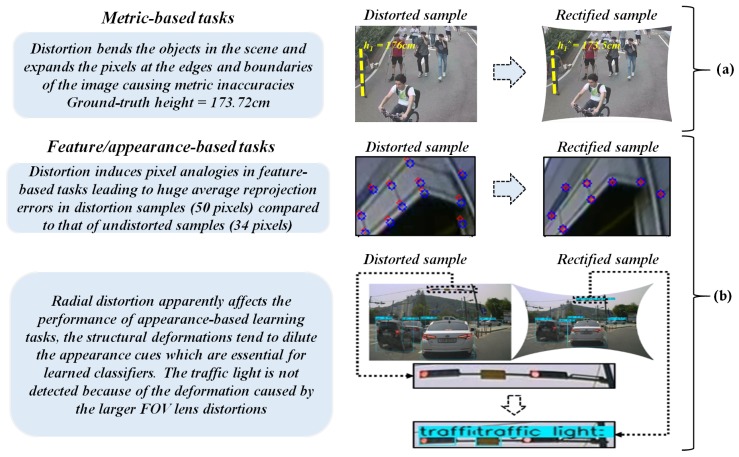
Effects of larger FOV distortions on high-end vision-based tasks. (**a**) Metric-based tasks and (**b**) feature/appearance-based tasks.

The image projections from the wide-angle and fish-eye lens are generally affected by the radial distortions and thereby create a scenario of severe pixel inconsistencies along the edges which depend on the properties of the lens such as horizontal FOV, curvature, etc. [1,2]. This indeed influences the performance of the lens employed in various metric-based tasks such as height estimation and single metrology, and even in geometrical tasks such as camera localization, stereo-vision, etc. This analogy can be observed in Figure 1, where a self-calibrated camera frame is used for streamlining various vision-based tasks in scenarios of diverse vision applications.

**Figure 2:**
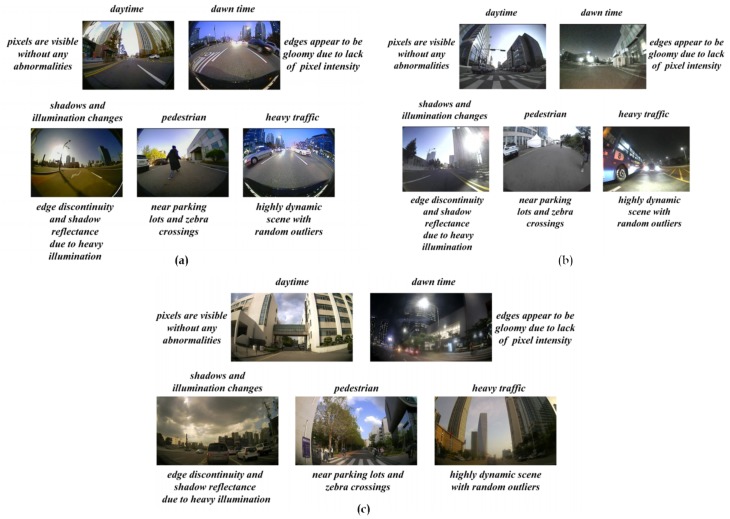
Real-time challenging scenarios: (**a**) fish-eye model (165${}^{\circ }$< FOV < 190${}^{\circ }$); (**b**) wide-angle (120${}^{\circ }$< FOV < 150${}^{\circ }$); (**c**) super wide-angle (160${}^{\circ }$< FOV < 180${}^{\circ }$).

The flexibility of handling diverse lens models is another major concern in the formulation of a robust self-calibration technique. The presence of various larger FOV lens models such as fish-eye (165${}^{\circ }$< FOV < 190${}^{\circ }$), wide-angle (120${}^{\circ }$< FOV < 150${}^{\circ }$), and super wide-angle (160${}^{\circ }$< FOV < 180${}^{\circ }$) impose severe challenges in determination of distortion parameters for each class and compensating the specific lens models automatically. The variations in lens models and real-time scenarios are depicted in Figure 2.

The fish-eye and wide-angle lens models are manufactured with a basic notion of the coverage area that the lens can capture. In accordance with that, the lens usually possesses severe distortions due to which the scene aspects on the image plane tend to deviate from the factual representation of a 3D real-world plane. Under such circumstances, the calibration is very important to retrieve the distortion-rectified scene while simultaneously preserving the automatic sense of adaptability without the involvement of any chessboards or objects. Self-calibration totally depends on the scene aspects such as lines, curves, points at infinity, edge candidates, special elements, etc. Several methodologies have been proposed to get past these challenges to formulate robust self-calibration techniques [3,4,5] but they still get severely caught off with inevitable real-world scenarios such as variation in illuminations, shadow castings, different timings in the day and night, and scenes with limited scene attributes to rely on.

### 1.2. Purpose of Study

The primary purpose of this work is to develop a flexible automatic distortion rectification methodology that can refine the outliers simultaneously, optimizing the best-fit parameters with minimum error possible. As an underlying investigation, the study has been incorporated by streamlining the distortion-rectified frames for acquiring better performance on tasks such as object detection and fixed monocamera-based height estimation. The two main aspects that this work clearly studies are how the proposed system can be robust towards various real-time scenarios with diverse challenges, and how the streamlining of vision tasks can be done with respect to the distortion-rectified frames. The main contributions are as follows: (1) Proposing an iterative optimization with refinement of the outliers from the pool of robust line-member set; (2) formulating plumbline angular cumulative loss over refined line-member set and investigating the significance through an ablation approach; (3) validating the proposed system with respect to quantitative (accuracy, processing time, practical significance) and qualitative (adaptability, practical significance) aspects on diverse real/synthetic, public and private datasets with respect to ADAS and video-surveillance applications. The scope of this study is targeting the high-end vision-based applications such as intelligent transportation, video surveillance, and advanced driver-assistant systems (ADAS).

The paper is organized as follows. Section 2 extensively discusses the previous works and their characteristics regarding the automatic distortion rectification. Section 3 elaborates on the proposed outlier refinement enabling the automatic distortion rectification process. Section 4 is dedicated to investigating the significance of proposed aspects with respect to various datasets and metrics. Section 5 illustrates the experimental design and evaluation metrics employed in the study. Section 6 reports the outcomes and corresponding discussions based on employed data and evaluations. Finally, Section 7 concludes the paper with a summary.

## 2. Literature Review

### 2.1. Automatic Distortion Rectification

In the literature, there are a plethora of studies that were designed to deal with radial distortion rectification via autocalibration of the camera systems [6,7,8]. Most of these simply followed the approach of employing the calibration object such as a checkerboard or circular patterns [9]. In practice, these camera systems tend to suffer from the variations in the weather conditions with respect to overheating or cold [10,11]. In situations as such, the calibration of the camera must be done to adjust the intrinsic parameters. Automatic distortion rectification, being a more practical approach, can come in handy in such circumstances. Especially, lens models such as fish-eye and wide-angle camera systems demand a better algorithm that can rectify the radial distortions.

Few works like Zhang et al. [12] and Barreto et al. [13] proposed their version of approaches in solving this problem through autocalibration of the visual sensor using scene attributes. However, their approaches demand a specific set of the environment such as precise structured lines (presence of at least three orthogonal straight lines). Brown et al. [14] was the first study to coin the term plumbline, specifying the usage of scene geometry for retrieving the camera’s intrinsic parameters. Additionally, this study specified the radial distortions using the polynomial lens distortion model. Later, the one-parameter rational model was proposed by [15,16] which were extensively used in the automatic camera calibration. In literature, the variants of plumbline approaches were used, among which employing of vanishing points to calibrate the camera yielded better results [17]. Yet, their approach was not able to handle wide-angle lens models with heavy distortions.

### 2.2. Previous Works

The automatic distortion rectification problem can typically be resolved using two main methodologies such as traditional and deep-learning approaches. In the traditional approach, various geometrical aspects are exploited to estimate the distortion parameters of the lens. On the other hand, deep-learning approaches estimate the distortion parameters through learned radial distortion values and image samples. Though there are various algorithms in the above two portfolios, there exist some limitations which make the algorithm venerable towards various real-world conditions.

In the past decade, few remarkable studies were proposed in the context of automatic rectification of wide-angle and fish-eye lens models. A few studies were formulated to explore the arithmetic approach on the line curvatures to estimate the distortions [4]. A few others exploited the scene lines to estimate the parameters with intense iterative optimizations [3,5] within parametric Hough spaces, and a few employed the semiautomatic algebraic approach of tracing line segments over the curved lines for estimating distortions [18]. The semiautomatic study proposed by Alvarez et al. [18] heavily requires user-interaction in the line tracing approach, which is not appropriate for real-time usage. Although Bukhari et al. [4] was able to rectify the distortions with reliable performance for nonsevere distortion cases, it suffers from longer processing times and deformed outputs in the case of heavy distortions. The Hough parametric space approaches from Aleman et al. [3] and Santana et al. [5] were able to rectify the wide-angle and fish-eye lens models with reasonable performance. However, the heavy dependency on hyper-parameters and disability to handle samples acquired using low-quality camera sensors under low-light conditions make it less reliable for ADAS and video surveillance applications. Although, the algorithm proposed by Kakani et al. [19,20] was able to rectify multiple lens models which include a wide-angle and fish-eye lens. Yet, the schematic includes model-specific empirical γ-residual rectification factor for heavy fish-eye distortions with FOV > 165${}^{\circ }$. The design of this factor requires a certain amount of prior knowledge about the lens models from an optical perspective.

CNN deep-learning approaches such as Bogdan et al. [21] and Lopez et al. [22] cannot rectify the distortion samples with illumination changes, and certain higher distortion ranges cannot be handled with consistency. Additionally, deep GANs such as Liao, Kang et al. [23] are used for generating corresponding rectified samples for a distorted image. Yet, the trained distortions are confined to certain ranges such as <−10${}^{-5}$. Another GAN-based architecture proposed by Park et al. [24] was able to rectify the synthetic distorted samples as well as real sensor data within a specified distortion range. However, in the context of heavy distortion ranges, the model fails to rectify the samples. The major concern regarding these learning approaches is that the training examples must cover almost all the sensor types and ranges of the distortions in order to develop a model that can best rectify all the possible sensor units. In reality, this is not quite possible with the currently available advancements. This raises an issue of using only a certain sensor type and distortion range for a specific application such that one can attain the best performance using learning-based methodologies on that sensor unit. This must be done with each and every sensor unit in correspondence to the use-case that has to be deployed on the rectified frames. Due to this ambiguity, the present proposed work ruled out the learned method in performance evaluations. The details of the summarized state-of-the-art automatic distortion rectification techniques are stated in Table 1.

This study focuses mostly on the drawbacks encountered in our previous work [19] and proposes a solution to handle heavy distortions without having to use any model-specific residual factors. Especially, this work introduces the outlier refinement scheme in conjunction with the plumbline angular loss function that makes the whole system more robust to outliers and thereby able to handle heavy distortions FOV > 190${}^{\circ }$. The significance of the novel aspects—such as loss aggregation over line-member sets—of the outlier refinement scheme was extensively tested through ablation study, and the corresponding results are discussed in Section 4. The major difference between our previous work [19] and the current study is as follows:

- The segregation of robust line candidates was done on the basis of threshold heuristics in the previous work [19], which made some outliers raise some complications while dealing with heavy distortions FOV > 165${}^{\circ }$, thereby creating a need for model-specific residual factors.
- Unlike [19], the current study employs an iterative outlier refinement scheme which basically considers the aggregation of robust line members into a set and iterating the sets over the plumbline angular loss constraint. The loss over the cumulative line-member sets and corresponding estimated distortion parameters are used to eliminate the outliers, thereby using the new set of robust line candidates to update parameters for distortion rectification.
- The current plumbline angular loss constraint with respect to optimization scheme is analogous to that of [19], but the optimization is altered to consider the loss over the cumulative line-member sets to estimate the distortion parameters with simultaneous outlier elimination.

## 3. Outlier-Refinement-Enabled Distortion Estimation

### 3.1. Lens Distortion Parameter Modeling

In this study, the distortion estimation and optimization procedures were followed as per the odd polynomial lens-distortion model with up to two distortion coefficients $D_{1},D_{2}$ as per the design in our previous work [19], which maps rectified pixel coordinates to the distorted pixel coordinates, as shown in Equation (1) below.

(1) $$\begin{array}{c} r_{dist}=r_{undist}+D_{1}\cdot r_{undist}^{3}+D_{2}\cdot r_{undist}^{5},\\ r_{dist}=r_{undist}\left(1+D_{1}\cdot r_{undist}^{2}+D_{2}\cdot r_{undist}^{4}\right), \end{array}$$

where $r\left(radius\right)=\sqrt{{\left(a-a_{0}\right)}^{2}+{\left(b-b_{0}\right)}^{2}}$, (a,b) is a point coordinate, $\left(a_{0},b_{0}\right)$ is the image center, and $D_{1},D_{2},\cdots D_{N}$ are distortion coefficients.

### 3.2. Plumbline Angular Loss Estimation

The plumbline angular loss is estimated on the robust line-member set, the line members are extracted using parameter-free edge drawing algorithm [25]. Line members emerging from the same edge sources are further filtered based on length threshold heuristics. The line-member set was formed with the elements as line members emerging from same edge. There exists several line-member sets which are to be considered to calculate the cumulative loss on a whole.

The image $I_{wxhx3}$ represents an image and $\bar{n}$ denotes the number of line-member sets within the image *I*. The collection of all line-member sets as a matrix $L_{\bar{n}\times 4}$, where each line-member set consists of several line members. Each line member is a 4-tuple $\left(x_{0},y_{0},x_{1},y_{1}\right)$, where $\left(x_{0},y_{0}\right)$ represent the starting point and $\left(x_{0},y_{0}\right)$ represent the ending points of the line member. The grouped line members are collected as

(2) $$l_{ki}=\left[\begin{array}{c} \left(x_{0,1},y_{0,1},x_{1,1},y_{1,1}\right)\\ \left(x_{0,2},y_{0,2},x_{1,2},y_{1,2}\right)\\ \vdots \\ \left(x_{0,k},y_{0,\bar{k}},x_{1,k},y_{1,k}\right) \end{array}\right],$$

(3) $$L_{\bar{n}\times 4}=\left[\begin{array}{c} l_{k1\times 4}\\ l_{k2\times 4}\\ \vdots \\ \vdots \\ l_{k\bar{n}\times 4} \end{array}\right],$$

where $k\in 1,2,\ldots .\bar{n}$, for instance, $l_{ki}=l_{23}$ indicates that this is the second line-member set and it consists of three line members.

The angular plumbline error α can be estimated through the function $A\left(l_{1},l_{2}\right)$ which computes the angular difference between the line members in a set as shown below:

(4) $$A\left(l_{1},l_{2}\right)=\left\{\begin{array}{c} \Delta \alpha \\ 360-\Delta \alpha \;\;\mathrm{if}\;\Delta \alpha >180 \end{array}\right.,$$

(5) $$\Delta \alpha =\left|\alpha_{1}-\alpha_{2}\right|,$$

(6) $$\alpha_{i}=\arctan2\left(y_{1}-y_{0},x_{1}-x_{0}\right).$$

The angular plumbline error α with respect to all *N* line members is estimated, and an individual line member errors LE for the *i*th element of the line-member set is calculated by applying cross-entropy of the angular plumbline error:

(7) $${\mathrm{LE}}_{i\times \bar{n}}=-\frac{1}{N}\left(\begin{array}{c} \left|\Delta \alpha_{i,i+1}\right|\log\left|\Delta \alpha_{i,i+1}\right|,\left|\Delta \alpha_{i,i+2}\right|\log\left|\Delta \alpha_{i,i+2}\right|,\ldots ,\\ \left|\Delta \alpha_{i,i+k}\right|\log\left|\Delta \alpha_{i,i+k}\right|,\left|\Delta \alpha_{i+1,i+2}\right|\log\left|\Delta \alpha_{i+1,i+2}\right|\\ \left|\Delta \alpha_{i+1,i+3}\right|\log\left|\Delta \alpha_{i+1,i+3}\right|,\ldots ,\\ \left|\Delta \alpha_{i+1,i+k-1}\right|\log\left|\Delta \alpha_{i+1,i+k-1}\right|,\ldots ,\\ \left|\Delta \alpha_{i+k-2,i+k-1+\bar{n}}\right|\log\left|\Delta \alpha_{i+k-2,i+k-1+\bar{n}}\right| \end{array}\right),$$

where $k=\left|LE_{i\times \bar{n}}\right|$, i.e, the length of *i*th row

(8) $$\mathrm{SE}=\left(\frac{\sum_{1}^{\left|LE_{1}\right|}{LE}_{1}}{\left|{LE}_{1}\right|},\frac{\sum_{1}^{\left|LE_{2}\right|}{LE}_{2}}{\left|{LE}_{2}\right|},\ldots ,\frac{\sum_{1}^{\left|LE_{\bar{n}}\right|}{LE}_{\bar{n}}}{\left|{LE}_{\bar{n}}\right|}\right),$$

where SE (line-member set errors) is a row vector of length $\bar{n}$, which represents the average of the *i*th line-member set.

The mean cumulative loss SMCE which computes the mean errors of a line-member set given by $L_{\bar{n}\times 4}$ as follows:

(9) $$\mathrm{SMCE}\left(L_{\bar{n}\times 4}\right)=\frac{\sum_{1}^{\left|SE\right|}SE}{\left|SE\right|},$$

where |SE| is the cardinal set of all line-member set errors.

This overall error loss must be minimized such that we can accomplish two things in one-shot:

- By minimizing error and refining the accumulated line-member set such that the unwanted curves and outliers in the image can be pruned.
- Additionally, through minimizing the error equation, we can estimate the distortion parameter.

### 3.3. Refinement Optimization Scheme

The Levenberg–Marquardt (LM) optimization, which was employed in the current study, estimates the best fit parameters with simultaneous outlier elimination, where the camera lens parameters are initial with default initial guess:

(10) $$\;\mathrm{Params}\;=\left[\begin{array}{c} f_{x}\\ f_{y}\\ c_{x}\\ c_{y}\\ D_{1}\\ D_{2} \end{array}\right].$$

Let $f_{x},f_{y},c_{x},c_{y},D_{1},D_{2}$ represent the focal length of *x* (in pixels), the focal length of *y* (in pixels), the *x* position of the camera center, the *y* position of the camera center, and the distortion parameters, respectively.

(11) $$r_{\bar{n}\times 1}=\left[\begin{array}{c} r_{1\times 1}\\ r_{2\times 1}\\ \vdots \\ \vdots \\ r_{\bar{n}\times 1} \end{array}\right];\;x_{\bar{n}\times 1}=\left[\begin{array}{c} x_{1\times 1}\\ x_{2\times 1}\\ \vdots \\ x_{\bar{n}\times 1} \end{array}\right];\;y_{\bar{n}\times 1}=\left[\begin{array}{c} y_{1\times 1}\\ y_{2\times 1}\\ \vdots \\ y_{\bar{n}\times 1} \end{array}\right],$$

where $r_{\bar{n}\times 1}^{\prime }$ is the column vector of radial distortions for each line member within the line-member set given by $L_{\bar{n}\times 4}$, and $r_{i\times 1}^{\prime }=\sqrt{x_{i\times 1}^{{}^{\prime }2}+y_{i\times 1}^{{}^{\prime }2}}$ in which $x_{i\times 1}^{\prime },y_{i\times 1}^{\prime }$ are the corresponding *x* and *y* coordinates of the *i*th radial distortion—$i\in \{1,2,\ldots \bar{n}\}$.

(12) $$x_{i}=\frac{x_{i}^{\prime }}{\left(1+D_{1}r_{i}^{2}+D_{2}r_{i}^{4}\right)},\;y_{i}=\frac{y_{i}^{\prime }}{\left(1+D_{1}r_{i}^{2}+D_{2}r_{i}^{4}\right)},$$

where undistorted $x_{i}$ and $y_{i}$ points are mapped using the distorted parameters $D_{1}$ and $D_{2}$ with respect to $r_{i}$, resulting in distorted points $x_{i}^{\prime }$ and $y_{i}^{\prime }$. In addition, $Pts_{1}={{\left[\begin{array}{c} \left.L_{\bar{n}\times \left[1,2\right]}\right]\\ L_{\bar{n}\times \left[3,4\right]} \end{array}\right]}_{2\bar{n}\times 2}}^{1}$ represent the matrix of undistorted start and end points of the line-member set.

(13) $${L_{\bar{n}\times \left[1,2\right]}}^{1}=x_{i}\times f_{x}+cx={\left[\begin{array}{c} \left(x_{0,1},y_{0,1}\right)\\ \left(x_{0,2},y_{0,2}\right)\\ \vdots \\ \left(x_{0,\bar{n}},y_{0,\bar{n}}\right) \end{array}\right]}^{1},$$

(14) $${L_{\bar{n}\times \left[3,4\right]}}^{1}=y_{i}\times f_{y}+cy={\left[\begin{array}{c} \left(x_{1,1},y_{1,1}\right)\\ \left(x_{1,2},y_{1,2}\right)\\ \vdots \\ \left(x_{1,\bar{n}},y_{1,\bar{n}}\right) \end{array}\right]}^{1}.$$

Let $L_{\bar{n}\times 4}$ represent a matrix for the set of line members of an image, where $l_{ki}$ is the matrix formed by all the line members. The overall mean cumulative line-member set error (SMCE) in the image is estimated using the initial parameters and line members ${L_{\bar{n}\times 4}}^{0}$:

(15) $${L_{\bar{n}\times 4}}^{0}=\left[\begin{array}{c} l_{k1\times 4}\\ l_{k2\times 4}\\ \vdots \\ l_{k\bar{n}\times 4} \end{array}\right];\;l_{ki}=\left[\begin{array}{c} \left(x_{0,1},y_{0,1},x_{1,1},y_{1,1}\right)\\ \left(x_{0,2},y_{0,2},x_{1,2},y_{1,2}\right)\\ \vdots \\ \left(x_{0,k},y_{0,k},x_{1,k},y_{1,k}\right) \end{array}\right].$$

The parameters are used to refine the outliers by eliminating unwanted set of line members with respect to minimum error and then an iterative process of elimination takes place to see if the error is getting minimized further by eliminating unwanted outliers *i*th line member and forming new line-member set $l_{(k-1)i}$ for distortion estimation as shown below:

(16) $${{\mathrm{Err}}_{L_{\bar{n}\times 4}}}^{0}=SMCE\left(D_{1},D_{2},I,{L_{\bar{n}\times 4}}^{0}\right);$$

(17) $$l_{(k-1)i}=\left[\begin{array}{c} \left(x_{0,1},y_{0,1},x_{1,1},y_{1,1}\right)\\ \left(x_{0,2},y_{0,2},x_{1,2},y_{1,2}\right)\\ \vdots \\ \left(x_{0,k-1},y_{0,k-1},x_{1,k-1},y_{1,k-1}\right) \end{array}\right].$$

Similarly, $L_{\bar{n},\left(j-1\right)\times 4}$ is the submatrix formed by removing the outliers and retaining j−1 line members from the *n*th line-member set; thereby, the error $Err_{L\bar{n},\left(j-1\right)\times 4}$ corresponding to the outlier refinement can be estimated simultaneously such that the sequence of submatrices $L_{\bar{n},\left(1\right)\times 4},L_{\bar{n},\left(2\right)\times 4},\cdots L_{\bar{n},\left(j-1\right)\times 4}$ and their corresponding line-member set errors ${\mathrm{Err}}_{L_{\bar{n},\left(1\right)\times 4}},{\mathrm{Err}}_{L_{\bar{n},\left(2\right)\times 4}},\ldots {\mathrm{Err}}_{L_{\bar{n},\left(j-1\right)\times 4}}$ are formed:

(18) $$\begin{array}{c} L_{\bar{n},\left(j-1\right)\times 4}=\left[\begin{array}{c} l_{k1\times 4}\\ l_{k2\times 4}\\ l_{k3\times 4}\\ \cdot \\ \cdot \\ l_{k(j-1)\times 4} \end{array}\right],\\ {\mathrm{Err}}_{L\bar{n},\left(j-1\right)\times 4}=SMCE\left(D_{1},D_{2},I,L_{\bar{n},\left(j-1\right)\times 4}\right). \end{array}$$

**Figure 3:**
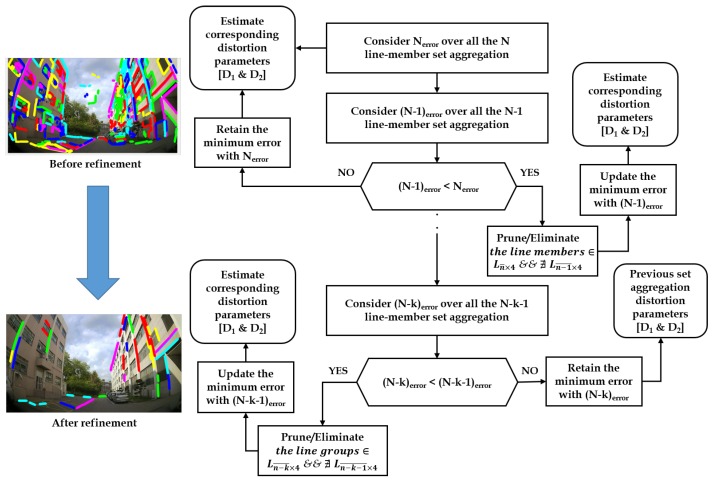
Outlier refinement scheme based on line-member set aggregations.

The final line-member sets containing refined line members with minimum error are elected for the distortion parameter estimation. The election process of robust line-member set (ELS) is depicted in the Figure 3.

(19) $$ELS=\left\{\begin{array}{cc} if\min\left(Err_{L\bar{n}\times 4},{\mathrm{Err}}_{L\bar{n},\left(j-1\right)\times 4}\right)=Err_{L\bar{n},\left(j-1\right)\times 4} & L_{\bar{n},\left(j-1\right)\times 4}\\ \mathrm{Otherwise} & L_{\bar{n}\times 4}, \end{array}\right.$$

where j∈{1,2,…i}; $i\in \{1,2,\ldots \bar{n}\}$.

## 4. Ablation Study

### Practical Significance Analysis

The ablation study serves as a practical significance analysis investigating the novel aspects introduced in this work. Additionally, this study differentiates the method using straightness loss constraint on individual line candidates [19] from the proposed method of cumulative set aggregation loss and refinement scheme. This investigation will assist in understanding the real significance of using these aspects in the proposed system and their influence on the output performance:

- **Quantitative:** Investigation of proposed cumulative set aggregation loss and refinement scheme with respect to image quality, edge stretching, pixel-point error, and processing time on distorted KITTI dataset and distortion center benchmark.
- **Qualitative:** Investigation of proposed cumulative set aggregation loss and refinement scheme with respect to real-time adaptability and feasible undistortion on severe distortions (FOV: 140${}^{\circ }$ and 165${}^{\circ }$) with respect to private CV Lab Larger FOV real dataset.

**Figure 4:**
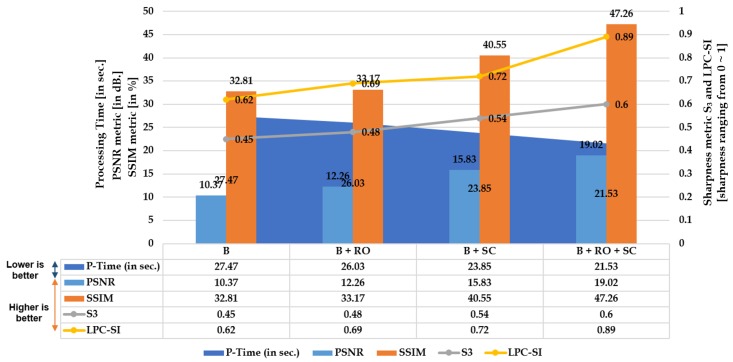
Quantitative: Significance of proposed cumulative set aggregation loss and refinement scheme with respect to image quality, edge stretching, and processing time on distorted KITTI dataset.

**Figure 5:**
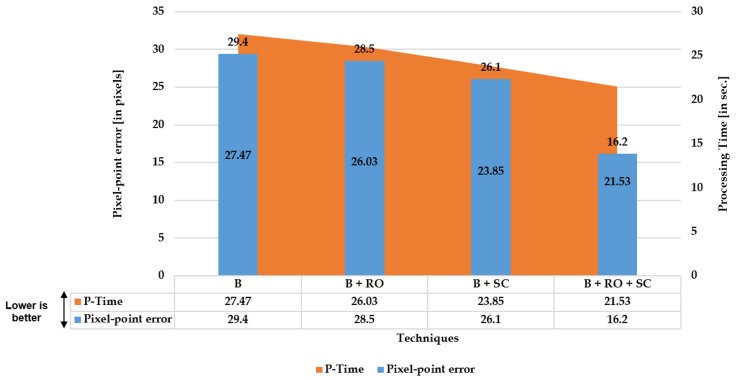
Quantitative: Significance of proposed cumulative set aggregation loss and refinement scheme with respect to pixel-point error and processing time on distortion center benchmark dataset.

**Figure 6:**
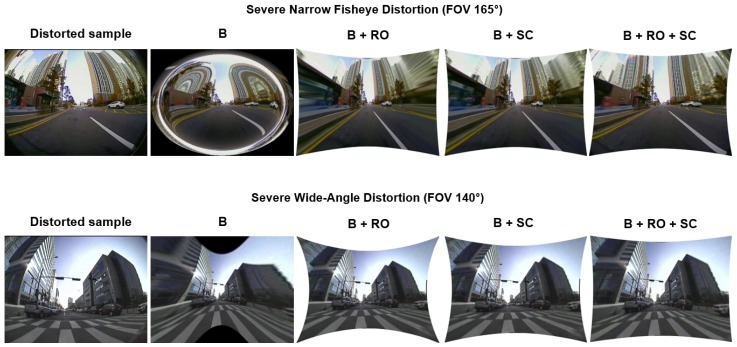
Qualitative: Significance of proposed cumulative set aggregation loss and refinement scheme with respect to severe distortions.

The three Figure 4, Figure 5 and Figure 6 illustrated below depict the quantitative and qualitative significance analysis of the proposed novel elements over various public and private datasets with respect to diverse metrics. The clear influence of the proposed elements such as cumulative set aggregation loss and refinement scheme can be observed in the qualitative analysis depicted in Figure 6. The following acronyms are used: B—Baseline; B + RO—Baseline + Refined optimization scheme; B + SC—Baseline + Set cumulative aggregation; B + RO + SC—Baseline + Refined optimization scheme + Set cumulative aggregation. Various combinations were used in the ablation study to mainly understand the practical significance of the proposed elements. The clear explanation of the combinations is as follows:

- **B:** Uses the basic straightness loss constraint between line members (without outlier refinement) to estimate distortion parameters.
- **B + RO:** Uses the basic straightness loss constraint between line members (with outlier refinement) to estimate distortion parameters.
- **B + SC:** Uses the basic straightness loss constraint over set cumulative line-member sets (without outlier refinement) to estimate distortion parameters.
- **B + SC + RO:** Uses the basic straightness loss constraint over set cumulative line-member sets (with outlier refinement) to estimate distortion parameters.

## 5. Experiments and Evaluations

### 5.1. Pixel Quality and Consistency Experiments

The experiments were carried out to examine the pixel quality and consistency of the rectified image and low-level image-quality metrics were considered accordingly. The synthetic distorted KITTI dataset using [26,27] was employed to evaluate the rectified image with respect to GT (distortion-free KITTI sample). The accuracy of the distortion-rectified image can be evaluated in two different ways such as image quality metrics, peak signal-to-noise ratio (PSNR); structural similarity index (SSIM); spectral, spatial, and sharpness metric ($S_{3}$); local phase coherence sharpness index (LPC-SI); and pixel consistency metrics such as pixel-point error (PPE). The subsections below illustrate the individual significance of each evaluation method present in both strategies.

#### 5.1.1. Image Quality Evaluations

The image quality of the distortion-rectified image must be preserved, and it can be validated using comparative measures with respect to original distortion-free samples in terms of similarly and noise aspects.

- Peak Signal-to-Noise Ratio (PSNR): The pixel consistency of the output (undistorted image) with respect to the original distortion-free image can be assessed using PSNR value. The mathematical measure is directly proportional to the quality of the output, i.e., if the PSNR value is high, the signal information in the output image corresponding to that of the distortion-free image is high and vice versa.
- Structural Similarity Index (SSIM): SSIM is one of the most prominent metrics, which is analogous to human visual perception. The fundamental blocks in the estimation of SSIM are luminance (*L*), contrast (*C*), and structural difference (*S*), which are calculated using the combinations of mean, standard deviation, and covariance [28].
- Spectral spatial sharpness ($S_{3}$): The $S_{3}$ metric was proposed by [29] and is best suited to examine the sharpness of an image without the reference ground truth. This metric can be retrieved from the pixel properties of the image in terms of spectral and spatial attributes. First, the color image is converted to grayscale and then $S_{1}$ and $S_{2}$ are extracted from the grayscale image. The metric $S_{1}$ represents the spectral sharpness map which is the local magnitude spectrum slope; and the metric $S_{2}$ represents the spatial sharpness map which is the local total variation. The geometric mean of these $S_{1}$ and $S_{2}$ is termed as final sharpness map $S_{3}$, which is the overall perceived sharpness of the entire image.
- Local phase coherence sharpness index (LPC-SI): This metric was introduced by [30] to evaluate the sharpness of an image from a different perspective rather than using edge, gradient, and frequency content. This sharpness metric quantifies the sharpness of an image with strong local phase coherence.

#### 5.1.2. Pixel-Point Error Evaluation

**Figure 7:**
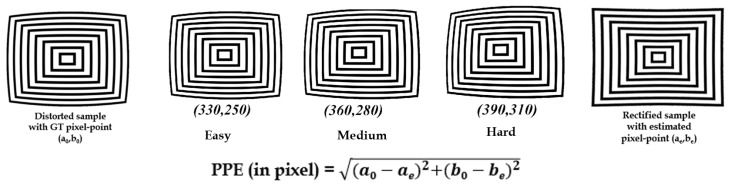
Pixel-point error calculation on distortion center synthetic dataset [4].

The pixel-point error was calculated by estimating the distance between the ground truth pixel point location and the refined image pixel point. For this experiment, the synthetic distortion center benchmark dataset [4] was utilized as shown in the Figure 7 below:

### 5.2. High-Level Metrics: ADAS and Video-Surveillance Experiments

This subsection elaborates on the essential usage of wide-angle and fish-eye lens models with proposed automatic distortion rectification techniques to yield better performance in the ADAS, video-surveillance-based vision tasks. In the ADAS context, the state-of-the-art (SOTA) pretrained models were employed to evaluate the proposed algorithm in terms of object detection on real and synthetic data. In the video-surveillance tasks, the height estimation using fixed camera intrinsics from [31] was employed to evaluate the proposed algorithm. The datasets used in this study were collected at Computer Vision Laboratory, Inha University, among which some are publicly available [31] and few were stated in our previous works [19].

#### 5.2.1. Datasets Used

The datasets utilized in the experiments were of three types:

- **Public-Synthetic dataset:** The publicly available KITTI dataset was synthetically modified using open-sourced distortion induction codes [26]. This dataset can be used to quantitatively measure the performance of distortion rectification algorithms and high-level metrics.
- **Private-Real dataset:** This dataset has been collected using various cameras with diverse lens models such as fish-eye (190${}^{\circ }$) and wide-angle (120${}^{\circ }$). This real dataset tests the robustness of the rectification algorithms with respect to the object detection scenarios.
- **Public- and Private-Real dataset:** This dataset has been collected using various cameras with diverse lens models such as super wide-angle (150${}^{\circ }$) and wide-angle (120${}^{\circ }$). This real dataset tests the robustness of the rectification algorithms with respect to the height estimation and metric-level information.

#### 5.2.2. Object Detection Using Pretrained Models

**Figure 8:**
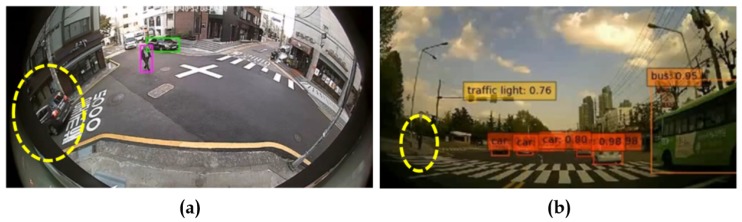
Performance of pretrained state-of-the-art (SOTA) models on different larger FOV raw samples: (**a**) Pretrained YOLOv3 on 190${}^{\circ }$ fish-eye sample (car undetected along the edge); (**b**) Pretrained SSD on 120${}^{\circ }$ wide-angle sample (person undetected along the edge).

Various pretrained models were employed, such as YOLOv3 (pretrained on PASCAL VOC) and SSD (pretrained on MS COCO), as object detectors. These experiments were carried out on diverse lens models such as fish-eye (190${}^{\circ }$) and wide-angle (120${}^{\circ }$). The qualitative comparisons were made between various automatic rectification algorithms with respect to detection along the edges. Additionally, for the quantitative measure, the distorted KITTI data samples are rectified using various algorithms alongside the proposed method, and the detection mean average precision (mAP) scores were recorded. The major intent of investigating the proposed algorithm against various algorithms on SOTA pretrained object detectors is to validate the improved performance on rectified frames in streamlining (deploying) object detection tasks. In normal raw samples, the detection accuracy drops due to the distortions along the edges and using SOTA object detectors on those frames would not help, as shown in Figure 8:

#### 5.2.3. Height Estimation on Fixed Monocamera Sensor

The height estimation is considered a metric-based task, as the pixel distribution in the image plays a vital role in deciding the metric information. For a fixed camera setup, the experiments were designed on the basis of estimating the intrinsic using walking humans metrology, proposed by Li, Shengzhe et al. [31], employing the Computer Vision Lab’s video-surveillance dataset collected at Inha University.

**Figure 9:**
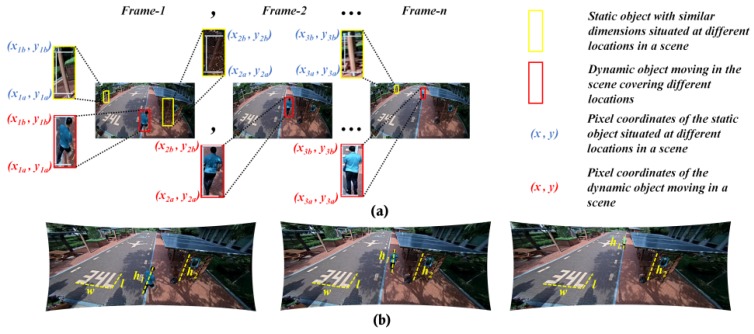
Retrieval of distortion-rectified reference pixel correspondences for better accuracy: (**a**) Top and bottom reference points in distorted case; (**b**) Corresponding top and bottom rectified reference points in rectified case.

During this study, we modified the previous height estimation method [31] such that the rectified pixel points are retrieved and used to initiate the pixel locations of the walking human (top and bottom) for intrinsic-based height estimation. The modified phenomenon is illustrated in Figure 9, where the objects are not deformed as they are in the raw distortion samples. The camera sensors used in evaluating the algorithm under this portfolio are wide-angle lens cameras. They are employed to capture all the data, as specified in [31], and the subjects used in that study were used in our study as well to maintain the consistency in the ground truth. The height estimation errors in cm is used as a metric for better comparison.

## 6. Results and Discussions

### 6.1. Pixel Quality and Consistency

**Figure 10:**
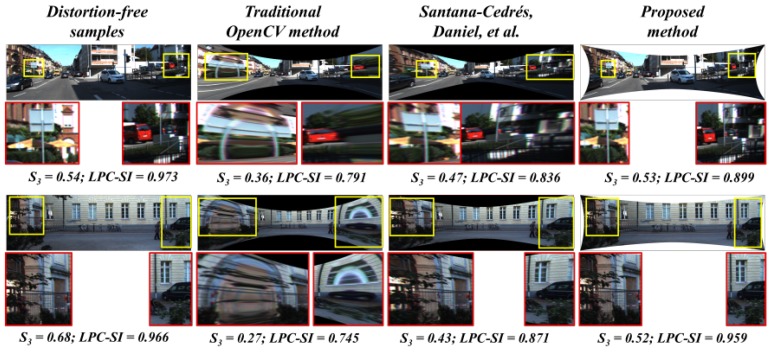
Qualitative analysis: pixel quality and consistency.

The consistency in the pixel information, especially regarding the stretching issue, was clearly investigated, as shown in Figure 10 below. The stretching along the edges caused the inconsistency in the case of traditional OpenCV and Santana et al. [5]. Due to the refinement of outliers, the stretching was significantly reduced in the proposed method.

#### 6.1.1. Quantitative Analysis: Image Quality

The proposed method was able to rectify the random synthetic distortions, and the average image quality scores in terms of similar metrics and spectral context seem to be high compared to that of the manual and automatic methods. The corresponding results are illustrated in Table 2.

#### 6.1.2. Quantitative Analysis: Pixel-Point Error

The pixel-point error calculations were made using difference of distances from two pixel points in the rectified image distortion center and given GT distortion center on difference samples. The average pixel-point errors were calculated against [5,18] algorithms and the results are stated in Table 3 below. The average pixel-point error in the case of Alvarez et al. [18] and Santana et al. [5] appears to be higher for the examples that have higher variations in the distortion center. The filtering of line-member set for robust line candidate selection influences the proposed method to attain lower average pixel-point error. For the better understanding of quantitative analysis, the average pixel-point errors of all the three methods are indicated in bold.

### 6.2. High-Level Metrics: ADAS Use-Case

The data samples utilized in the experiments were mainly ADAS-centered and are heavily distorted in terms of field-of-view and real-time challenges. The performance analysis was carried out both qualitatively and quantitatively against various automatic distortion rectification methodologies.

#### 6.2.1. Qualitative Performance Analysis

**Figure 11:**
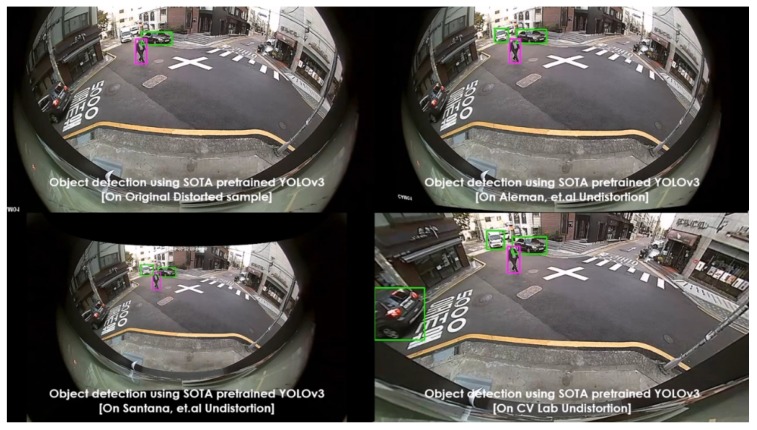
Pretrained YOLOv3 object detection on various rectified 190${}^{\circ }$ fish-eye frames: car detected along the edge in the proposed rectified algorithm.

**Figure 12:**
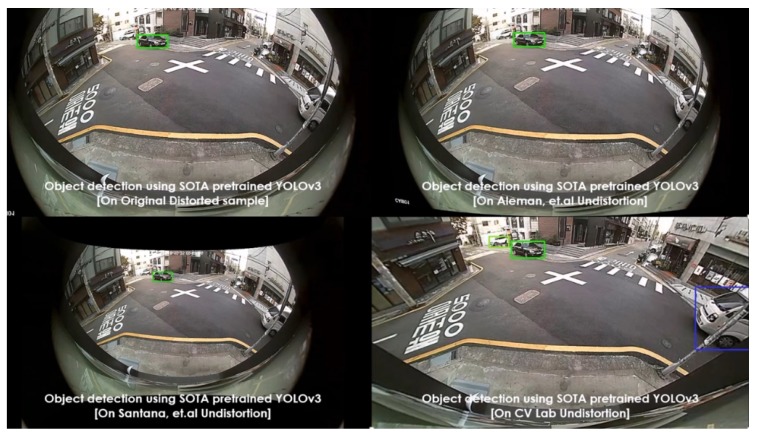
Pretrained YOLOv3 object detection on various rectified 190${}^{\circ }$ fish-eye frames: van detected along the edge in the proposed rectified algorithm.

**Figure 13:**
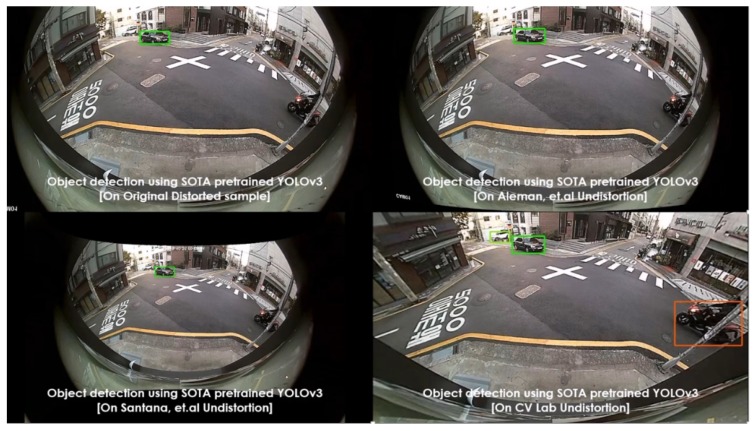
Pretrained YOLOv3 object detection on various rectified 190${}^{\circ }$ fish-eye frames: motorbike detected along the edge in the proposed rectified algorithm.

**Figure 14:**
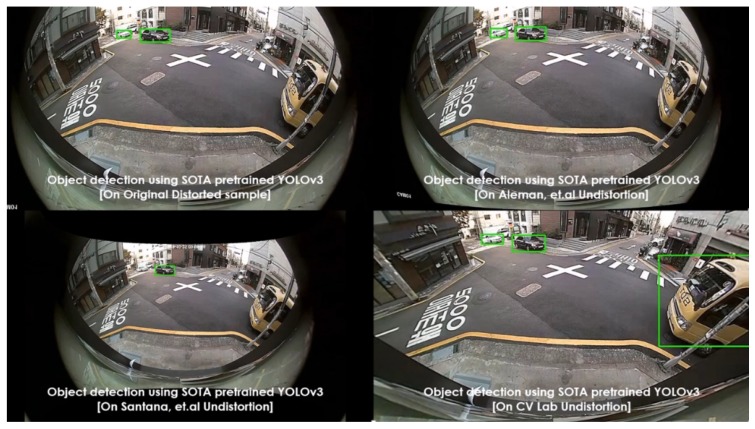
Pretrained YOLOv3 object detection on various rectified 190${}^{\circ }$ fish-eye frames: bus detected along the edge in the proposed rectified algorithm.

**Figure 15:**
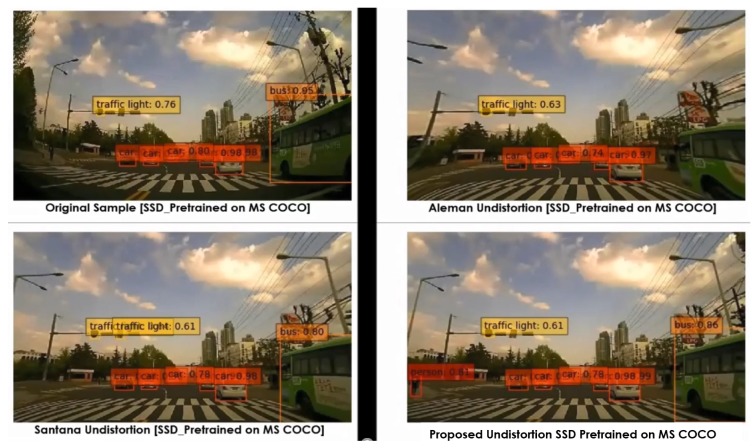
Pretrained SSD object detection on various rectified 120${}^{\circ }$ wide-angle frames: person detected in the proposed rectified algorithm frame.

The performance comparisons were carried out between original samples, Aleman et al. [3], Santana et al. [5], and the proposed method with respect to two pretrained models on 3 different cameras. The results were depicted in Figure 11, Figure 12, Figure 13, Figure 14 and Figure 15 to illustrate the case-by-case scenario robustness of object detection. The objects such as person, car, truck, motorbike, and bus were successfully detected in the case of rectified samples using the proposed method. Although the same pretrained detector was employed on all the SOTA-rectified frames, the proposed method frame yields best performance.

#### 6.2.2. Quantitative Performance Analysis

**Figure 16:**
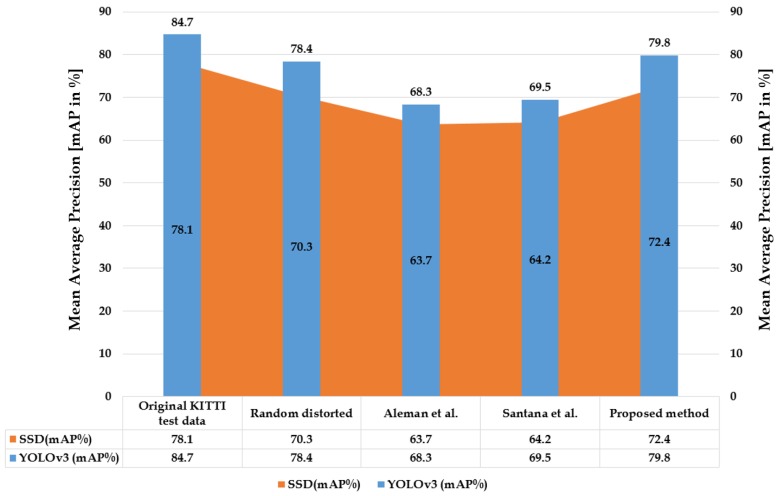
SOTA pretrained YOLOv3 and SSD were employed to detect the objects in the scene on distorted KITTI samples rectified with various algorithms.

The quantitative analysis has been carried out using the synthetic distorted KITTI dataset on various rectified algorithms—Aleman et al. [3], Santana et al. [5], and the proposed method—alongside distortion-free and randomly distorted samples. The SOTA pretrained YOLOv3 and SSD were employed to detect the objects in the scene, and comparisons were done with respect to various cases. The corresponding quantitative analysis in terms of mAP is depicted in Figure 16. The pretrained SSD achieved 72.4 mAP on rectified samples using the proposed method, which is higher than the distorted an other rectified samples. Similarly, pretrained YOLOv3 achieved 79.8 mAP on proposed method rectified samples, which is greater than the distorted and other rectified samples. The rectified samples used in the streamlining of trained detectors must perform well in order to improve the detection accuracy, and this must be validated using distortion-free samples for proper analysis. The original samples are considered as a ground-truth benchmark such that the algorithm which can produces better rectified samples can therefore be streamlined on to pretrained detectors for better accuracy. This phenomenon proves that the rectified samples using the proposed method are more pixel-consistent and preserved the object characteristics through stretch-free rectification compared to the other rectification algorithms.

### 6.3. High-Level Metrics: Video-Surveillance Use-Case

The quantitative and qualitative analysis was carried out on various samples retrieved from different camera systems. Primarily, the comparisons were carried out between the use cases where the inevitability of distortion is high. Both the quantitative and qualitative analyses were dealt with using experiments where the distortions were rectified and thereby the intrinsic estimation and height calculations were performed. This process was done for both cases—the distortion rectification process proposed in this study as well as the manual rectification following the approach of Li, Shengzhe et al. [32]. The accuracy in height measurements was estimated with a straightforward method of retrieving errors between the estimated and available ground truth.

**Figure 17:**
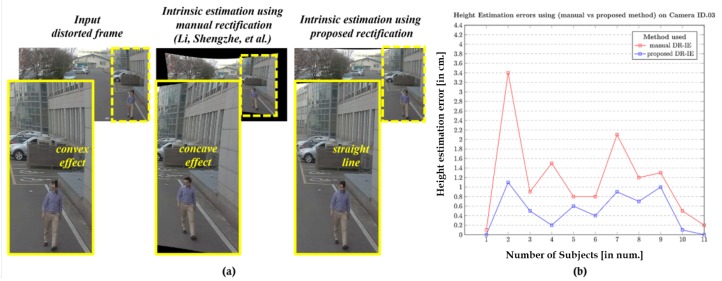
Height Estimation errors using (Li, Shengzhe et al. [31] vs. proposed method) on Outdoor camera ID.03: (**a**) Qualitative pixel-consistency. (**b**) Height estimation error plot corresponding to all the 11 subjects.

**Figure 18:**
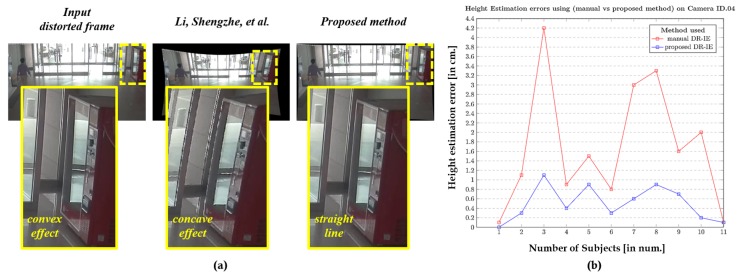
Height Estimation errors using (Li, Shengzhe et al. [31] vs. proposed method) on Indoor camera ID.04: (**a**) Qualitative pixel-consistency. (**b**) Height estimation error plot corresponding to all the 11 subjects.

**Figure 19:**
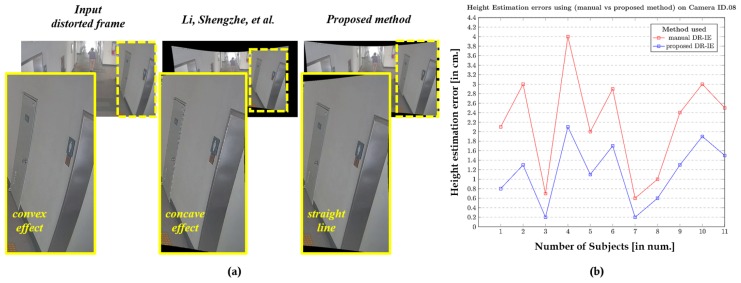
Height Estimation errors using (Li, Shengzhe et al. [31] vs. proposed method) on Indoor camera ID.08: (**a**) Qualitative pixel-consistency. (**b**) Height estimation error plot corresponding to all the 11 subjects.

The results corresponding to the camera IDs 03, 04, and 08 are depicted in Figure 17, Figure 18 and Figure 19, respectively, as they spread-over the samples retrieved from both indoor and outdoor. The distortion effect was nullified using both the rectification methods, and the rectified pixel points were used for the further process of estimating the heights of all 11 subjects recorded using a similar camera ID. The red plot line represents the height error values in the case of manual rectification, where the distortions are not completely rectified and that resembles a concave effect due to inappropriate estimation of distortion parameters. The blue plot line represents the error in height estimations in case of the rectification using proposed method.

The results clearly state that the method used in Li, Shengzhe et al. [32] is manual in a manner with the intrinsic-based height estimation, which can be termed as manual distortion-rectification-guided intrinsic-based height estimation (DR-IE) has an effect due to pixel irregularities. This inconsistency in pixel locations and corresponding error in metric information increases with the increase in the distortion levels. The method proposed by Li, Shengzhe et al. [32] is unable to handle such irregularities through manual rectification. In contrast, the proposed method uses the rectified frames to get the pixel location which has relatively low pixel inconsistency resulting in the low height estimation error in cm. This can be clearly shown in the error plots where the height estimation errors are relatively larger in Li, Shengzhe et al. [32] than the proposed method.

**Figure 20:**
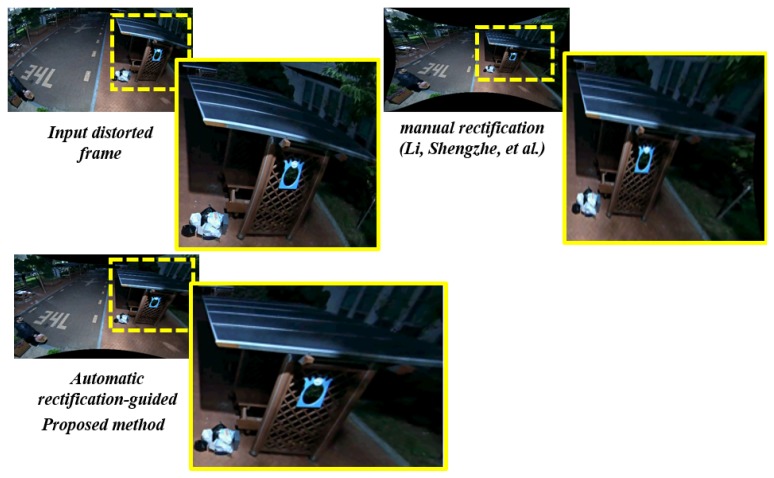
Robustness of proposed distortion-rectification-guided height estimation on wide-angle camera at night time.

The effect of the distortion-rectification-guided height estimation can be observed clearly in the context of the wide-angle camera scenario. The below Figure 20 illustrates the robustness of the proposed system in the presence of darkness and severe illumination changes.

The overall height estimation errors with respect to various camera sensors in the context of 11 subjects have been extensively tested with the Li, Shengzhe et al. [31] result as a baseline. The proposed method preserved the pixel consistency in the distortion-rectified image, thereby when those rectified pixels are used for the height estimations, the errors seem to decline. These quantitative comparisons are clearly illustrated in Table 4 below. The camera IDs 1, 2, 6, 7 were used to compare the distortion effects on the metric height estimation because these camera sensors posses a slightly higher amount of distortions compared to the other camera sensors used in the study. The average height estimation errors are indicated in bold in the below table which clearly explains the effectiveness of height estimation via the proposed automatic distortion rectification method.

## 7. Conclusions

An outlier refinement methodology for automatic distortion rectification of wide-angle and fish-eye lens camera models was proposed. The novel cumulative plumbline angular loss over line-member set aggregation exhibits better performance in conjunction with the outlier refinement optimization scheme. The design elements were evaluated using various metrics on real datasets (wide-angle: 120${}^{\circ }$< FOV < 150${}^{\circ }$; fish-eye: 165${}^{\circ }$< FOV < 190${}^{\circ }$) and synthetic distortions on distorted KITTI comprising of several real-time challenges and diverse distortion variations. The practical significance of the proposed novel elements was investigated using an ablation study in accordance with public and private datasets on image quality and pixel consistency metrics. The novel cumulative plumbline angular loss in conjunction with outlier refinement optimization scheme exhibited better performance in rectifying severe distortions compared to other rectification options in the ablation study. A diverse range of experiments were conducted in relevance to the low-level metrics such as image quality, stretching, and pixel-point error on various metrics such as PSNR, SSIM, S3, and LPC-SI. Besides, most of the experiments were carried out in the context of streamlining vision tasks on the rectified frames. The high-level scenarios, such as object detection in ADAS and metric height estimation in video surveillance, were extensively exploited on the distortion-rectified frames to validate the proposed method. Application-oriented metrics such as mean average precision (mAP) and height estimation errors (in cm) were employed to investigate the adaptability of the proposed method in both learning-based appearance tasks and metric-based tasks. Both the quantitative and qualitative metrics were employed in all the streamlined experiments to examine the practical usage of the proposed method. The rectification algorithm proposed using the outlier refinement optimization scheme guided the streamlining vision-based tasks to achieve better accuracy.

## Figures and Tables

**Table 1:** Insights of traditional and learning-based automatic distortion rectification methods.

Algorithm	Method	Dataset	Limitations
Alvarez et al. [18]	2D Euclidean distance	Synthetic dataset with symmetrical patterns	Semiautomatic. Not robust for real-time usage (illumination changes, etc.)
Bukhari et al. [4]	Circular arcs algebra	Synthetic dataset with salient point GT	Severe stretching along the edges. Long processing time for heavy distortion samples
Aleman et al. [3]	Hough parametric space	A private dataset using Nikon D90	Unstable outputs for larger FOV lens camera samples. Heavy hyperparameter-dependent
Santana et al. [5]	Iterative optimization of Hough transforms	Wide angle lens distortion image	Lacking robustness towards blurred images and low-light conditions
Kakani et al. [19]	Straightness cost constraint loss with model-specific empirical γ-residual rectification factor	Real data with varying distortion ranges 120${}^{\circ }$< FOV < 200${}^{\circ }$ Synthetic distorted KITTI samples	Requires prior model-specific knowledge to deal with γ-residual rectification factors
Bogdan et al. [21]	Dual CNN network on radial distortions	Panoramic images of the SUN360 dataset	Fails to rectify samples in illumination changes, motion blur samples
Lopez et al. [22]	CNN Parameterization for radial distortions	SUN360 panorama dataset	Network can only undistort in cropped mode rising an issue of pixel loss ≥30%
Park et al. [24]	U-Net-based GAN for radial distortions	Real and synthetic distortion dataset	Cannot handle heavy distortions FOV > 160${}^{\circ }$
Liao, Kang et al. [23]	U-Net-based GAN for radial distortions	Synthetic dataset with distortion ranging	Limited distortion ranges (cannot handle distortions <$-10^{-5}$)

**Table 2:** Qualitative analysis: image quality metrics on synthetic distorted dataset.

Image Quality Metrics	Distortion Rectification Algorithm			
	Traditional OpenCV	Bukhari et al. [4]	Santana et al. [5]	Proposed Method
PSNR [in dB.]	8.75	13.61	17.5	19
SSIM [in %]	22.9	30.3	43.2	47.2
S3 [↓0∼1↑]	0.44	0.34	0.41	0.51
LPC-SI [↓0∼1↑]	0.78	0.82	0.86	0.92

**Table 3:** Quantitative analysis: pixel-point error metrics on synthetic distorted dataset.

Synthetic Distortion Pixel-Point (GT)	Pixel-Point Errors on Distortion-Rectified Samples [in px.]		
	Alvarez et al. [18]	Santana et al. [5]	Proposed Method
Easy (330,250)	21.3	14.1	10.1
Medium (360,280)	17.0	18.4	15.9
Hard (390,310)	49.5	39.9	28.8
**Average point error [in px.]**	**29.2**	**24.1**	**18.3**

**Table 4:** Quantitative comparison: Average height estimation errors with respect to various cameras on 11 subjects.

Subject ID	Height Estimation Errors with Respect to Various Cameras on 11 Subjects [in cm]							
	Cam1		Cam2		Cam6		Cam7	
	Manual	Automatic	Manual	Automatic	Manual	Automatic	Manual	Automatic
S1	0.1	0	0.1	0.2	0.1	0	0.1	0.1
S2	1	0.4	2	0.7	0.5	0.2	0.1	0.2
S3	0.1	0	0.2	0.1	1.2	0.7	0.6	0.5
S4	0.1	0	0.8	0.4	1.3	0.9	2.2	0.4
S5	4.2	1.5	0.2	0.3	3	0.6	3	1.2
S6	0.5	0.3	1.4	0.5	0.4	0.2	2.4	0.8
S7	2.6	0.7	3	0.8	0.3	0.2	0	0.1
S8	1.1	0.9	0.9	0.6	1.2	0.7	1.1	0.7
S9	2	0.6	0.3	0.2	0.4	0.3	0.9	0.8
S10	4.1	1.1	0.9	0.3	0.8	0.1	2.1	0.6
S11	2.8	1.3	1.3	0.2	1.4	0.4	0.8	0.5
**Average** **Errors [in cm]**	**1.69**	**0.61**	**1.01**	**0.39**	**0.96**	**0.39**	**1.20**	**0.53**
